# The Generative Mechanism of Boomerang Intention: From the Perspective of Legacy Identification

**DOI:** 10.3389/fpsyg.2021.807887

**Published:** 2022-01-24

**Authors:** Zehui Tian, Qinghong Yuan, Shanshan Qian, Yanyan Liu

**Affiliations:** ^1^Business School, Nankai University, Tianjin, China; ^2^Business School, Guangdong University of Foreign Studies, Guangzhou, China

**Keywords:** boomerang employment, boomerang intention, legacy identification, organizational prestige, psychological contract violation

## Abstract

Boomerang employment has become an increasingly significant third way to obtain employees, yet little research has focused on why does ex-employee want to come back. Drawing from social identity theory, we propose that legacy identification could increase boomerang intention and both perceived corporate prestige and psychological contract violation could affect boomerang intention through legacy identification. The cooperative relationship between the former organization and the current organization could enhance these effects. Results from a two-time points survey of 202 Chinese employees showed that legacy identification could increase boomerang intention, perceived corporate prestige could increase boomerang intention via legacy identification, psychological contract violation could decrease boomerang intention via legacy identification. Besides, the positive effect of legacy identification on boomerang intention, the positive indirect effect of corporate prestige on boomerang intention via legacy identification, and the negative indirect effect of psychological contract violation on boomerang intention via legacy identification are all stronger when there is a cooperative relationship. Theoretical and practical implications are discussed.

## Introduction

Historically, voluntary turnover is regarded as a betrayal and is largely considered to be the end of the relationship between the employee and the organization. When an individual “terminates” his/her current employment relationship, he/she cuts off the connection with their former organization forever (Shipp et al., 2014). But this judgment ignores the possibility that ex-employees may return to the company in the future, which is called “boomerang employment” (Arnold et al., 2021). Besides, technological, societal, and economic changes are accelerating the tendency for employees to switch employers (Mawdsley and Somaya, 2016). Thus a large scale of ex-employees appears (Laulié and Morgeson, 2021), providing numerous human resources for the former organization. As we know, internal and external hiring have their limitations (Dlugos and Keller, 2021), while re-hiring has advantages over them in some aspects. Such as improving the accuracy of employee selection; reducing the time and cost of recruitment, training, and socialization; bringing new knowledge, skills, and perspectives to the organization; boosting the morale for the existing employees; enhancing customer retention; strengthening employees’ trust and commitment to the organization after they have found that “the pasture outside is not greener”, and then establishing long-term employment relations (Shipp et al., 2014; Raveendra and Satish, 2021). More and more companies are willing to tear down these “bad labels” that have been put on their ex-employees and welcome them to return. Some companies even provide a more convenient way for ex-employees to return, without going through a complex formal recruitment process and may maintain their previous salary and position levels (Arnold et al., 2021). At the same time, some ex-employees are also willing to be re-employed, which can be mainly divided into two categories: Planned boomerang employees are usually related to non-work factors such as life cycles (for example, leaving the company temporarily to balance work and family, and subsequently returning when the problem disappears over time), while unplanned ones are often associated with cognitive misjudgments (for example, the new firm is not as good as expected, a return to the former organization after discovering that the former is better) (Shipp et al., 2014). Data shows that the proportion of re-hired employees can reach approximately 20% (Shipp et al., 2014), and this proportion shows a growing trend over time (Swider et al., 2017). In conclusion, ex-employees have increasingly become a third human resource source of organizations (Arnold et al., 2021).

Based on the planned behavior theory (Ajzen, 1985), boomerang intention is the most immediate and significant predictor of ex-employee’s boomerang behavior (Raveendra and Satish, 2021). However, little attention has been paid to this topic. However, most of the related studies focused on the attitude, behavior, and performance of boomerang employees after being rehired. Specifically, Arnold et al. (2021) studied the job performance and turnover rate of the ex-employees after they have been rehired; Snyder et al. (2021) showed the psychological contract reconstruction and the resulting improvement in compensation, satisfaction, organizational commitment, extra-role/in-role behaviors, and performance of boomerang employees after they have been rehired; Maier et al. (2021) showed that boomerang employees who have returned to a previous employer consider their new situation better than the first time; Swider et al. (2017) extend a careers-based learning perspective to construct a theoretical framework of a set of factors that influence boomerang employee return performance. Although Shipp et al. (2014) and Raveendra and Satish (2021) focused on the antecedents and processes before the rehiring. The former showed some key drivers for the previous employer to hire ex-employees and the boomerang hiring process from the perspective of the previous employer; the latter identified original tenure and turnover style as influencing factors of return back. However, research on how the boomerang intention generates is limited, although it means a lot to boomerang employment (Raveendra and Satish, 2021).

Social identity theory plays a significant role in explaining an individual’s intentions and behaviors which are group-related (Hogg, 2006, 2021). According to previous studies, members can still identify the organization they do not belong to anymore, which is called “legacy identification” (Eury et al., 2018). Social identity theory holds that individuals are likely to choose and perform activities consistent with their identification (Ashforth and Mael, 1989). Thus as ex-employees still identified with their former organizations, their legacy identification may increase their boomerang intention, which plays a significant role in explaining how boomerang intention generates. According to social identity theory, self-enhancement and positive distinctiveness are the significant motivations of identification (Hogg, 2006). Since the image of the organization to which they belong is closely related to their self-concept or identity, in other words, the attributes people use to define the organization will also define themselves (Hogg and Terry, 2000; Pratt et al., 2021). Positive organizational attributes then may enhance its member’s identification. Since organizational prestige is a way for organizations and their members to show their positive intergroup distinctiveness, we’re going to talk about perceived corporate prestige as an antecedent of legacy identification in this study. Hogg (2006) also suggests that in addition to the self-classification we have talked about before. Social identity theory also speaks about the relationship quality between the organization and its members, which is the psychological base for the formation of social identification. Since the psychological contract is significant in the employee-organization relationship (Herrera and Las Heras-Rosas, 2021; Pandey and Pandey, 2021), this study also discusses psychological contract violation as an antecedent. In addition, for identification to affect intention, it must be psychologically salient (Hogg, 2006). In other words, everyone identifies with multiple organizations, and these identifications have varied degrees of salience in a given situation. Only when this identification becomes salient does it have an impact on subsequent intentions (Marin et al., 2009). This is particularly significant in the context of post-employment studies where legacy identification tends to be suppressed by their current organization. The legacy identification salient, which can be measured by the cooperative relationship between the former organization and current organization, moderates the relationship between legacy identification and boomerang intention. That is because the competitive relationship between the former organization and the current organization will make them hostile toward each other (Vaznyte et al., 2021). This study enriched the boomerang employment literature by focusing on the generative mechanism of boomerang intention.

**FIGURE 1:**
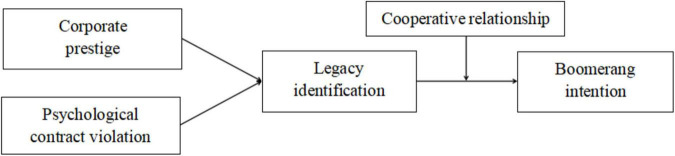
Hypothesized research model.

Based on the social identity theory, this study discusses the generation mechanism of boomerang intention. First, legacy identification was identified as an explanatory factor of the boomerang intention, providing us with a new perspective to explain ex-employee’s boomerang intention; second, based on social identity theory, the antecedents are explored, enriching the research on antecedents of boomerang intention; third, it pays attention to the post-employment context, identifying identity salient as the cooperative relationship between the current organization and former organization, thus specifying the boundary in the post-employment context. Figure 1 depicts our overall research model.

## Theoretical Basis and Hypothesis Development

### Legacy Identification and Boomerang Intention

The basic idea of social identity theory is that a social category into which one falls, and to which one feels one belongs, defines who he/she is in terms of the characteristics of their social category (Hogg and Terry, 2000). Legacy identification is derived from social identity theory, Eury et al. (2018) developed this concept to capture the way those members of an organization maintain, in the present, part of their self-definition from their past. That is because, first, Self-verification mechanism, which means that information consistent with people’s existing self-identities receives more attention, is better recalled, and is interpreted as more reliable, and that people often try to find or create social contexts that provide self-verifying feedback (Shrauger, 1975); second, the motivation of keeping the “self” stable, which means that people tend to maintain the stability of the “self” through maintaining a sense of “continuity across time and situation” within their identity (Vignoles et al., 2006). Based on that, identification can persist even after the relationship between the individual and the organization has ended. Although legacy identification is the continuity of organization identification, it does not necessarily preclude change. It’s a constant process of being remade according to the change of the environment (Walsh and Glynn, 2008; Gerstroem, 2015; Eury et al., 2018). Therefore, legacy identification is different from organizational identification in connotation and degree.

Social identity theory holds that individuals tend to perform activities consistent with their social identification (Ashforth and Mael, 1989). Since legacy identification refers to the ex-employees’ identification of their former organizations, it tends to develop intentions consistent with their former organization. In detail, via legacy identification, ex-employees perceive their identities to be at one with their former organization, and they tend to perceive their former organization’s purpose, characteristics, and values to be their own (Eury et al., 2018). So it is consistent with their legacy identification to be a member of their former organization again. Boomerang intention refers to the willingness of ex-employees to return to their former organizations. Therefore legacy identification may enhance boomerang intention. Findings of some researches can provide several supports, for example, nostalgia, as a significant legacy identification stratagem, represents a longing for the “good old days” they have had in their former organization, which could represent a willingness to return (Bardon et al., 2015). Thus, we propose the following hypothesis:

H1: Ex-employees’ legacy identification is positively related to their boomerang intention.

### Perceived Corporate Prestige, Psychological Contract Violation, and Legacy Identification

Based on social identity theory, self-enhancement and positive distinctiveness are the significant motivations of identification. Since the image of the organization to which they belong is closely related to their self-concept or identity, in other words, the attributes people use to define the organization will also define them (Hogg and Terry, 2000). Positive organizational attributes may enhance its member’s identification.

Dutton and Harquail (1994) propose that responses to the question “How do outsiders think of me because of my association with this organization?” And social identity theory could make an explanation, that is, the image of the organization to which they belong is closely related to their self-concept or identity, the attributes people use to define the organization will also define them (Hogg and Terry, 2000). In other words, self-concept or social identity is defined and evaluated in group terms (Hogg, 2006). Thus one of the most distinctive features of group life and intergroup relations that social identity theory has proposed is the positive distinctiveness—a belief that “we” are better than “them” in every possible way. As we know, perceived corporate prestige refers to the individual’s beliefs about outsiders’ perceptions of the organization (Shenkar and Yuchtman-Yaar, 1997), which is people’s evaluation of whether the corporate is good or not. When the external image is perceived as attractive, organizational affiliation creates a positive social identity for the individual, who is then motivated to increase his or her cognitive connection with the organization. In other words, the perceived corporate prestige of their former organizations can affect their legacy identification.

Some researches have provided supports for this relationship. March and Simon (1958) held that an individual identifies with a group partly to enhance self-esteem, the more prestigious the organization is, the greater the potential to boost self-esteem through identification, this research suggests that individuals’ propensity to identify with an organization increases with the organization’s perceived prestige. Vardi et al. (1989) demonstrated that the identification of organization members producing for the military market (a socially desirable role) is higher than those of a similar firm producing for the commercial market. Furthermore, Eury et al. (2018) found that when a former organization’s prestige is threatened by a scandal, the legacy identification held by its ex-employees can be challenged. Thus, we propose the following hypothesis:

H2a: Perceived corporate prestige is positively related to legacy identification.

At the same time, Hogg (2006) points out that the social identity approach doesn’t speak only about self-classification, and attaches much more importance to social interaction and interdependence. He believes that interaction, communication, and interdependence are bases for identification. Legacy identification is a process in which ex-employees psychologically belong to the organization. Therefore, when discussing the antecedents of legacy identification, we should not only focus on self-classification and its self-enhancement result, but also pay attention to the psychological basis of legacy identification. At the foundation of the employee-organization relationship is the psychological contract, comprised of beliefs about reciprocal obligations between the two parties (Morrison, 2000). When one party fails to fulfill its obligations, the psychological contract violation happens. Since the obligation performance is the only premise that determines if the psychological contract was violated or not (Rousseau, 1989), a break of employment contract often does not coincide with the break of the psychology contract. The psychological contract may endure even if the employment contract has broken down, and that would serve as a psychological base of legacy identification. Yet when the ex-employee feels that the organization had “failed to fulfill the obligation” in the employment tenure, or the turnover process, psychological contract violation happens (Robinson and Rousseau, 1994). That would trigger a strong negative emotional response, such as betrayal, disappointment, frustration, anger, or resentment (Morrison and Robinson, 1997), which may influence ex-employee’ s subsequent legacy identification. That is what makes the legacy identification psychologically real. Thus, we propose the following hypothesis:

H2b: Psychological contract violation is negatively related to legacy identification.

### The Mediating Role of Legacy Identification

According to social identity theory, both self-classification and interaction can affect legacy identification. First, social identification refers to the self-image derived from the social category to which they belong (Hogg, 2006). Membership to a social group (or social category) can enhance or decrease the self-esteem of its members, and its members tend to enhance the positive one, so perceived corporate prestige has a positive impact on legacy identification. Second, we should not only focus on social classification and self-enhancement but also pay attention to the psychological basis of legacy identification (Hogg, 2006). Psychological contract violation will decrease the legacy identification. Third, since individuals tend to develop intentions consistent with their social identification (Ashforth and Mael, 1989), legacy identification will influence subsequent boomerang intention of ex-employees. Thus, according to H1, H2a, and H2b, the following hypotheses are proposed:

H3a: Legacy identification mediates the relationship between perceived corporate prestige and boomerang intention.
H3b: Legacy identification mediates the relationship between psychological contract violation and boomerang intention.

### The Moderating Role of Cooperative Relationship

For identification to affect intention, it must be psychologically salient (Hogg, 2006). Identification salience refers to the possibility of an identification being awakened in a specific context (Hogg and White, 1995). In other words, everyone has multiple identifications (Serpe, 1994), and these identifications have varied degrees of salience in a given situation, only when legacy identification becomes salient does it influence subsequent intentions (Marin et al., 2009; Wood and Caldas, 2009). Identification salience has different connotations in different contexts, such as customer’s identification salient of a specific company (Marin et al., 2009), gender identification salient (Randel, 2002), and have been measured in different ways. As the cooperative relationship between the current organization and the former organization is a significant factor affecting the relationship between the ex-employees and their former organization (Somaya et al., 2008), legacy identification salient can be measured by the cooperative relationship between the former organization and the current organization in the context of post-employment. This is because, in situations with cooperative relationships, identifying with their former organization has higher legitimacy in their current organization due to the possibility of producing social benefits such as business cooperation, so the legacy identification is salient. The relationship between legacy identification and boomerang intention is stronger. Otherwise, identifying with their former organization is not encouraged by their current organization. Even is seen as a threat or betrayal to the current organization (Somaya et al., 2008; Bardon et al., 2015). So even if the ex-employee identifies strongly with their former organization, this legacy identification tends to be suppressed as they are working in their current organization, the relationship between legacy identification and boomerang intention is weaker. Therefore, the hypothesis is proposed:

H4: The cooperative relationship between the current organization and the former organization moderates the relationship between legacy identification and boomerang intention. Such that the positive relationship is stronger when there is a cooperative relationship.

According to H1, H2a, H3a, and H4, the following hypothesis is proposed:

H4a: The cooperative relationship between the current organization and the former organization moderates the relationship between perceived corporate prestige and boomerang intention through legacy identification. Such that the mediating effect is stronger when there is a cooperative relationship.

According to H1, H2b, H3b, and H4, the following hypothesis is proposed:

H4b: The cooperative relationship between the current organization and the former organization moderates the relationship between psychological contract violation and boomerang intention through legacy identification. Such that the mediating effect is stronger when there is a cooperative relationship.

## Materials and Methods

### Samples and Procedures

The Snowball sampling approach was used in this study to recruit participants, which is a suitable way for this study to obtain more heterogeneous data and improve the external validity (Lin et al., 2021). Direct participants were recruited through 4 universities’ alumni networks in China, and they were asked to recommend eligible participants. We explained the research purpose and procedures to voluntary leavers across different organizations, occupations, and locations in China. To facilitate the recruiting process, participants who completed each wave of the survey were promised to receive 5 RMB compensation.

We then distributed surveys at two-time points via WeChat. 297 participants provided demographic information, perceived corporate prestige, psychological contract violation, and cooperative relationship between former and current organizations at Time 1. Two weeks after the Time 1 survey, 237 participants provided ratings on legacy identification and boomerang intentions at Time 2. Moreover, to ensure data quality, we used participants’ WeChat ID (which was unique) to match the two-time point of surveys and avoid repetitive participation. We also used three questions for attention check (e.g., “For this item, please select ‘1’ “) to exclude inattentive respondents. After removing those mismatch surveys and inattentive data, the final sample size was 202, resulting in an overall response rate of 68.01%.

Of all the participants in this study, 53.1% were female; 63.8% were under the age of 30, and 28.1% were aged between 30-40; the average organizational tenure in their former company was 2.5 years (SD = 1.97); the average organizational tenure in their current company was 1.67 years (SD = 1.88); the average time lapse after living their former company was 1.69 years (SD = 1.87); As for the educational background, 68.8% has got a bachelor’s degree, and 20.8% has got a Master degree or above.

### Measures

Following Brislin’s (1980) “back translation” procedure, two professional people in our research field were invited to translate the scales into the Chinese version. One of them was responsible for translating the English items into Chinese, then the other was responsible for translating these items back into English. Through comparison and discussion, the Chinese version scales were obtained. Participants rated the extent to which they agree with each item on a 5-point Likert scale (1 = strongly disagree to 5 = strongly agree).

#### Perceived Corporate Prestige

We measured perceived corporate prestige using the seven-item scale developed by Mael and Ashforth (1992) in time 1. Items were adapted to the post-employment context. For example, “People around me think my former company is very well”. The Cronbach’s alpha was 0.88.

#### Psychological Contract Violation

We measured psychological contract violation using the four-item scale developed by Priesemuth and Taylor (2016) in time 1. Items were adapted to the post-employment context. For example, “I feel betrayed by my former organization”. The Cronbach’s alpha was 0.87.

#### Legacy Identification

We measured legacy identification using the 6-item scale developed by Mael and Ashforth (1992) in time 2. Although this scale is called the organizational identification scale originally, it has been widely used in measuring the legacy identification of alumni and ex-employees (Iyer et al., 1997). Items were adapted to the post-employment context. For example, “When someone praises my former company, I feel like I’m being praised”, The Cronbach’s alpha was 0.91.

#### Boomerang Intention

We measured boomerang intention using the 3-item scale in time 2. Hinkin’s (1998) method was used for item generation and verification.

First, the inductive approach is adopted in the step of item generation. 110 MBA students who have voluntary turnover experience were asked to provide descriptions of their feelings about their intention of getting back to their former organization. 75 of which were classified into 3 categories by 20 Ph.D. students in business school using the Q-Sorting technique with an agreement index of 75%. The remaining 35 responses were used to prove that these 3 items have reached theoretical saturation. Thus 3 items were generated (“I am willing to go back to my former company”; “I would feel happy if I had the chance to return to my former company”; “Among many employment opportunities, I prefer to go back to my former company”).

Second, 30 naive respondents were recruited for assessing content validity. Specifically, provided them with the definition of boomerang intention, and asked them to judge whether these 3 items corresponded with this definition. The result showed that all items were left according to an agreement index of 75%.

At last, data were collected from 98 valid respondents for initial item reduction. The results of exploratory factor analysis showed that all items were loaded on a single appropriate factor clearly, and all the factor loading is higher than 0.40. All the items were retained. The Cronbach’s alpha was 0.89. We measured this variable in time 2.

#### Cooperative Relationship

The cooperative relationship between the former and current organization is a categorical variable, measured by the question “Is your current company a cooperative company with your former company?” with the answer “yes” or “no”. We measured this variable in time 2.

#### Control Variables

In line with previous studies (Mael and Ashforth, 1992; Iyer et al., 1997), we controlled the organizational tenure, time-lapse, present tenure, and some demographic variables. We measured these variables by asking, “How long had you been working for your former company”, “How long have you been away from your former company”, and “How long have you been working for your current company”. The demographic variables include gender, age, education, and industry.

## Results

### Confirmatory Factor Analysis, Common Method Biases Test and Descriptive Statistics

Confirmatory factor analysis was conducted for the examination of the validities of the measures in our study. As presented in Table 1, the hypothesized 4-factor model (i.e., perceived corporate prestige, psychology contract violation, legacy identification and boomerang intention) yielded a better fit than alternative models (CMIN = 349.57, DF = 167, CMIN/DF = 2.09, RMSEA = 0.07, CFI = 0.92, TLI = 0.90, IFI = 0.93). All scale items loaded on their intended factors significantly (p < 0.001), ranging from0.55 to 0.99 for perceived corporate prestige, 0.78 to 0.96 for psychology contract violation, 0.75 to 0.96 for legacy identification, and 0.92 to 0.98 for boomerang intention.

**TABLE 1:** Confirmatory factor analysis for discriminant validity.

Factor structure	CMIN	DF	CMIN/DF	RMSEA	CFI	TLI	IFI
Four-factor model	349.57	167	2.09	0.07	0.92	0.90	0.93
Three-factor model	820.65	168	4.89	0.14	0.73	0.66	0.73
Two-factor model	1163.94	169	6.89	0.18	0.58	0.48	0.59
One-factor model	1369.94	170	8.06	0.18	0.50	0.38	0.56

*N = 202, The four-factor model includes perceived corporate prestige, psychological contract violation, legacy identification, and boomerang intention. The three-factor model includes perceived corporate prestige + psychological contract violation, legacy identification and boomerang intention. The two-factor model includes perceived corporate prestige + psychological contract violation + legacy identification and boomerang intention; The single factor model includes perceived corporate prestige + psychological contract violation + legacy identification + boomerang intention.*

There may be common method bias since the data was collected from a single source, which may lead to common method bias. In order to reduce the common methods biases, we take some measurements in the research process design, such as arranging questionnaire items randomly, collecting data anonymously, involving some reverse answer questions, and setting a number of screening questions. In addition, we conducted Harman’s s one-factor test to make an estimation. The result showed that the variance of one factor accounts for 27.27% of the total variance. Podsakoff and Organ (1986) contend that the proportion of method variance in total variance is about 50%. Therefore, it can be judged that there is no serious common method bias in this study.

The means, standard deviations, and correlations are presented in Table 2. Perceived organizational prestige was positively related to age (*r* = 0.21, *p* < 0.01), education (*r* = 0.14, *p* < 0.05) and former tenure (*r* = 0.19, *p* < 0.01), psychological contract violation was negatively related to gender (*r* = −0.14, *p* < 0.05), boomerang intention was positively related to age (*r* = 0.15, *p* < 0.05), therefore we controlled for their effect. Besides, organizational prestige was positively related to legacy identification (*r* = 0.43, *p* < 0.001) and boomerang intention (*r* = 0.43, *p* < 0.001), psychological contract violation was negatively related to legacy identification (r = −0.33, *p* < 0.001) and boomerang intention (r = −0.36, *p* < 0.001), cooperative relationship was positively related to boomerang intention (r = 0.18, *p* < 0.05). These results provide rudimentary support for our hypotheses.

**TABLE 2:** Means, standard deviations, and intercorrelations.

	Mean	SD	1	2	3	4	5	6	7	8	9	10
(1). Gender	1.56	0.50										
(2). Age	2.20	0.97	−0.30***									
(3). Education	3.86	0.61	0.03	0.07								
(4). Former tenure	2.50	1.98	–0.13	0.59***	0.16*							
(5). Time-lapse	1.69	1.87	−0.14*	0.43***	–0.06	0.35***						
(6). Present tenure	1.67	1.88	–0.13	0.40***	0.05	0.30***	0.76***					
(7). Perceived organizational prestige	3.42	0.75	–0.08	0.21**	0.14*	0.19**	–0.09	–0.07	(0.88)			
(8). Psychological contract violation	2.74	1.22	−0.14*	0.14	–0.03	0.05	0.11	0.09	−0.28***	(0.87)		
(9). Legacy identification	3.32	0.90	–0.02	0.11	0.08	0.08	–0.01	–0.06	0.43***	−0.33***	(0.91)	
(10). Cooperative relationship	0.79	0.41	0.05	0.02	–0.02	–0.01	–0.08	–0.05	0.14	−0.15*	0.15*	(0.91)
(11). Boomerang intention	2.39	1.08	0.06	0.01	0.15*	0.09	–0.02	–0.05	0.43***	−0.36***	0.50***	0.18*

N = 202, *p < 0.05. **p < 0.01. ***p < 0.001 (two-tailed). Gender was coded “1” for men and “2” for women. Education was coded “1” for “high school diploma or below,” “2” for “college diploma,” “3” for “bachelor degree” “4” for “master degree or above.”

### Hypothesis Test

Hierarchical regression analysis was conducted using SPSS 22.0 to test Hypothesis 1, 2a, and 2b. As we can see in Table 3, Model 9 showed that after controlling for age, gender, education, former tenure, time-lapse, and current tenure, legacy identification was positively associated with boomerang intention (β = 0.43, *p* < 0.001), supporting Hypothesis 1. Model 2 showed that after controlling for age, gender, education, former tenure, time-lapse, and current tenure, perceived corporate prestige was positively associated with legacy identification (β = 0.43, *p* < 0.001), supporting Hypothesis 2a. Model 3 showed that after controlling for age, gender, education, former tenure, time-lapse, and current tenure, psychological contract violation was negatively associated with legacy identification (β = −0.35, *p* < 0.001), supporting Hypothesis 2a.

**TABLE 3:** Results of multiple regression analysis.

	Legacy identification			Boomerang intention							
	Model 1	Model 2	Model 3	Model 4	Model 5	Model 6	Model 7	Model 8	Model 9	Model 10	Model11
Gender	0.05	0.02	–0.03	0.06	0.07	0.06	0.02	0.03	0.05	0.05	0.05
Age	0.14	0.04	0.18*	–0.01	–0.12	–0.13	0.03	–0.05	–0.08	–0.09	–0.08
Education	0.08	0.04	0.07	0.15	0.10	0.09	0.14*	0.11	0.11	0.11	0.12*
Former tenure	0.02	–0.03	–0.00	0.09	0.05	0.06	0.08	0.08	0.09	0.09	0.07
Time-lapse	0.06	0.14	0.08	0.05	0.13	0.08	0.07	0.04	0.02	0.04	0.03
Current tenure	–0.17	–0.14	–0.17	–0.11	–0.08	–0.03	–0.11	–0.04	–0.03	–0.03	–0.03
Perceived corporate prestige		0.43***			0.44***	0.28***					
Psychological contract violation			−0.35***				−0.36***	−0.22**			
Legacy identification						0.38***		0.42***	0.49***	0.48***	0.48***
Cooperative relationship										0.11	0.14**
Legacy identification * Cooperative relationship											0.14**
R-square	0.03	0.17	0.12	0.04	0.21	0.33	0.17	0.31	0.27	0.28	0.30
*F* value	1.11	6.66***	5.01***	1.29	7.44***	11.87***	5.47***	10.91***	10.36***	9.58***	9.24***

N = 202, *p < 0.05. **p < 0.01. ***p < 0.001 (two-tailed).

### Mediation Effect Results

Hypothesis 3a proposed that legacy identification mediates the relationship between perceived corporate prestige and boomerang intention. Model 4 of Hayes’ (2013) PROCESS macro with a bias-corrected bootstrapping procedure (5,000 repetitions) is used for the testing of hypothesis 3a. Results in Table 4 demonstrated that perceived corporate prestige was associated with boomerang intention indirectly through legacy identification (β = 0.24, SE = 0.06, bias-corrected 95% CI [0.14, 0.35]), providing support for Hypothesis 3a. Hypothesis 3b is tested using the same method. Results in Table 4 demonstrated that psychology contract violation was associated with boomerang intention indirectly through legacy identification (β = −0.12, SE = 0.03, bias-corrected 95% CI [−0.19, −0.03]), providing support for Hypothesis 3b.

**TABLE 4:** Results of mediation effect of legacy identity.

	Effect	Path	SE	Effect	95%CI	
					LLCI	ULCI
Perceived corporate prestige	Total effect	ALL	0.10	0.63	0.44	0.82
	Direct effect	Perceived corporate prestige → Boomerang intention	0.10	0.40	0.21	0.59
	Indirect effect	Legacy identification	0.05	0.23	0.13	0.34
Psychological contract violation	Total effect	ALL	0.06	–0.32	–0.44	–0.20
	Direct effect	Psychological contract violation → Boomerang intention	0.06	–0.19	–0.30	–0.08
	Indirect effect	Legacy identification	0.03	–0.13	–0.20	–0.07

*N = 202, bootstrap sample n = 5000; LLCI Lower limit of 95%CI; ULCI upper limit of 95%CI.*

### Moderation Effect Results

**FIGURE 2:**
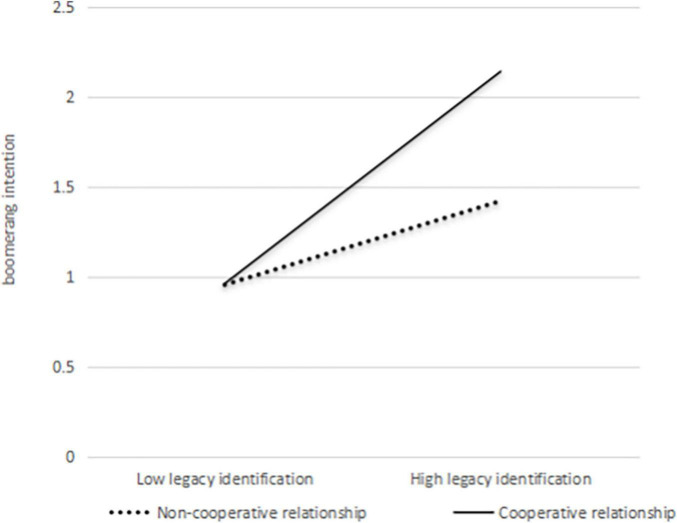
Moderating effect of cooperative relationship on the relationship between legacy identification and boomerang intention.

To test Hypothesis 4, we examined the interactive effect of legacy identification and cooperative relationship on boomerang intention. We tested it using hierarchical regression analysis with SPSS 22.0. Firstly, control variables, legacy identification, and cooperative relationship were entered into the regression equation. Then, the interaction term of legacy identification and cooperative relationship was entered. Legacy identification and cooperative relationship were mean-centered before the calculation of the interaction term (Aiken et al., 1991). Results in Model 11 of Table 3 showed that the interactive effect of legacy identification and cooperative relationship on boomerang intention was significant (β = 0.14, *p* < 0.05). As shown in Figure 2, simple slope tests showed that the relationship between legacy identification and boomerang intention was positive and significant when there is a cooperative relationship between the current and former organizations (β = 0.65, SE = 0.08, *p* < 0.001), but was not significant when there is a non-cooperative relationship (β = 0.26, SE = 0.16, *p* = 0.10 > 0.05).

### Moderated Mediation Results

Hayes’s (2013) PROCESS macro with 5,000 bias-corrected bootstrapped samples were used to test hypotheses 4a and 4b. The results are shown in Table 5. At the cooperative relationship level, perceived corporate prestige influenced boomerang intention via legacy identification in a significant way (β = 0.0.27, SE = 0.06, 95%CI [0.15, 0.40]), while at the non-cooperative relationship level, perceived corporate prestige’s effect on boomerang intention through legacy identification was not significant (β = 0.06, SE = 0.08, 95%CI [−0.10, 0.24]). The index for moderated mediation was 0.21 (SE = 0.10, 95% CI [0.01, 0.40]). Together, the findings suggest that the cooperative relationship between the current organization and the former organization moderates the relationship between perceived corporate prestige and boomerang intention through legacy identification, such that the mediating effect is stronger when there is a cooperative relationship, supporting Hypothesis 4a.

**TABLE 5:** Results of the moderated mediating effect.

Effect	SE	Effect	95%LLCI	95%ULCI
**Direct effect:Corporate prestige→Boomerang intention**				
Perceived corporate prestige→Boomerang intention	0.10	0.40	0.20	0.59
**Indirect effect:Perceived corporate prestige→Legacy identification→Boomerang intention**				
**Non-Cooperative** Corporate prestige → Legacy identification → Boomerang intention	0.16	0.12	–0.19	0.42
**Cooperative** Corporate prestige → Legacy identification → Boomerang intention	0.09	0.53	0.36	0.70
**Direct effect:Psychological contract violation→Boomerang intention**				
Psychological contract violation→Boomerang intention	0.06	–0.20	–0.32	–0.09
**Indirect effect:Psychological contract violation→legacy identification→boomerang intention**				
**Non-Cooperative** Psychological contract violation → legacy identification → boomerang intention	0.04	–0.03	–0.12	–0.05
**Cooperative** Psychological contract violation → legacy identification → boomerang intention	0.04	–0.15	–0.23	–0.08

*N = 202, bootstrap sample n = 5000; LLCI Lower/upper limit of 95%CI.*

Hypothesis 4b was tested in the same way. At the level of cooperative relationship, psychological contract violation had a significant indirect effect on boomerang intention through legacy identification (β = −0.15, SE = 0.04, 95%CI [−0.24, −0.08]), At the level of non-cooperative relationship, perceived corporate prestige had a weaker indirect effect on boomerang intention through legacy identification (β = −0.03, SE = 0.04, 95%CI [−0.12, −0.05]). The index for moderated mediation was −0.13 (SE = 0.06, 95% CI [−0.24, −0.03]). Together, the findings suggest that the cooperative relationship between the current organization and the former organization moderate the relationship between psychological contract violation and boomerang intention via legacy identification, such that the mediating effect is stronger when there is a cooperative relationship, supporting Hypothesis 4b.

## Discussion

### Research Conclusions

Based on the social identity theory and through data analysis, this study developed and tested a moderated mediation model to explain the generation mechanism of the boomerang intention of ex-employee. The conclusions are as follows: first, perceived corporate prestige has a significant positive impact on legacy identification. Second, psychological contract violation has a significant negative impact on legacy identification. Third, legacy identification plays a mediating role in the relationship between perceived corporate prestige and boomerang intention. Fourth, legacy identification plays a mediating role in the relationship between psychological contract violation and boomerang intention. The fifth, cooperative relationship between the current organization and former organization plays a moderating role in the relationship between legacy identification and boomerang intention. Sixth, cooperative relationship moderates the positive indirect effect between perceived corporate prestige and boomerang intentions through legacy identification, such that the mediated effect is stronger when there is a cooperative relationship. Similarly, the cooperative relationship moderates the negative indirect effect between psychological contract violation and boomerang intentions through legacy identification, such that the mediating effect is stronger when there is a cooperative relationship.

### Theoretical Implications

Since rehiring is an under-observed recruitment method in research, there is still an extreme lack of study about this topic (Arnold et al., 2021). Moreover, most related studies focused on the attitude, behavior, and performance of boomerang employees after being rehired (Swider et al., 2017; Arnold et al., 2021; Maier et al., 2021; Snyder et al., 2021). Although Shipp et al. (2014) and Raveendra and Satish (2021) focused on the antecedents and processes before the rehiring. The former was from the perspective of the previous employer; the latter only identified original tenure and turnover style as influencing factors of return back. However, research on how the boomerang intention generates is limited, although it means a lot to boomerang employment (Raveendra and Satish, 2021). Therefore, Several theoretical implications are made in this study. First, legacy identification is identified as an effective perspective to explain the generation mechanism of boomerang intention. Specifically, legacy identification is originated from social identity theory (Eury et al., 2018). The basic idea of social identity theory is that the social category into which one falls, and to which one feels one belongs, defines who he/she is in terms of the characteristics of their social category (Hogg and Terry, 2000) and that identification could affect people’s attitudes, intentions and behaviors (Hogg, 2006, 2021). Via legacy identification, ex-employees perceive their identities to be at one with their former organization. So it is consistent with their legacy identification to be a member of their former organization again. Thus legacy identification plays a significant role in illustrating the generation mechanism of boomerang intention.

Second, identify the antecedents of boomerang intention based on social identity theory. First of all, the base of social identity theory is social classification, one of the most distinctive features is the positive distinctiveness—a belief that “we” are better than “them” in every possible way. When the external image of the former organization is perceived as attractive, organizational affiliation creates a positive social identity for the individual, who is then motivated to increase his/her cognitive connection with the organization. Since perceived corporate prestige refers to an individual’s beliefs about outsiders’ perceptions of the organization, it is the influencing factor of boomerang intention. Besides, Hogg (2006) points out that the social identity approach doesn’t speak only about the social classification, and attaches much more importance to social interaction and interdependence between the ex-employee and their former organization. Psychological contract violation is significant to the relationship between ex-employees and their former organization. When an ex-employee feels that the organization had “failed to fulfill the obligation” in the employment tenure, or the turnover process, psychological contract violation happens (Robinson and Rousseau, 1994). That would trigger a strong negative emotional response (Morrison and Robinson, 1997), which may decrease ex-employees’ legacy identification and boomerang intention. Our study identified the two significant antecedents of boomerang intention based on social identity theory, revealing the antecedents of boomerang intention in a relatively completed way.

Third, identification salient is defined and measured differently in different situations, such as customer’s identification salient in the context of marketing (Marin et al., 2009), or gender identification salient in group conflict (Randel, 2002). Legacy identification salient can be measured by the cooperative relationship between the former organization and the current organization in the context of post-employment. In situations with cooperative relationships, identifying with their former organization has higher legitimacy in their current organization due to the possibility of producing social benefits such as business cooperation. The legacy identification is salient, the relationship between legacy identification and boomerang intention is stronger. Considering the cooperative relationship between the former organization and current organization as a moderating variable, not only enhance the explanation of the relationship between legacy identification and boomerang intention but also the development of the concept of identification salience in the context of post-employment, filling the vacancy of the literature in post-employment context.

### Practical Implications

The organizational practice of boomerang employment is increasingly popular and has many benefits. Some companies recognize the benefits and are willing to hire their ex-employees, but they do not have a reasonable plan to attract former employees to return. This paper calls on organizations to pay attention to developing this recruitment method and provides practical implications on how to increase the boomerang intention of ex-employees. First, perceived organizational prestige can influence the boomerang intention through legacy identification. Thus the organization should pay much attention to enhancing its prestige. For example, undertaking social responsibilities actively (Farooq et al., 2016), avoiding organizational scandal (Eury et al., 2018), hiring highly regarded CEOs since they are the face of the firm (Love et al., 2017), and taking appropriate measures to deal with necessary downsizing (Love and Kraatz, 2009). Through which provide some positive distinctiveness to their ex-employees, and then increase ex-employees’ legacy identification and boomerang intention.

Second, this study showed that the psychological contract violation can influence the boomerang intention through legacy identification. The organization should maintain a good relationship with the employees before and after their resignation. Especially in the process of exit. This process may play an important role in helping the employee and employer to transition from a formal employment relationship to a different kind of relationship (Klotz and Bolino, 2016), which may reprocess and update these impressions of their employers (Kulik et al., 2015). The human resources department needs to deal with it properly and cautiously since it will decrease legacy identification and boomerang intention. For example, avoiding wrongful termination claims (Lind et al., 2000), managing the resignation style (Klotz and Bolino, 2016), training in how to conduct effective exit conversations (Kulik et al., 2015), among others.

Third, this study found that the cooperative relationship between the current organization and the former organization positively moderates the relationship between legacy identification and boomerang intention, the relationship between perceived corporate prestige and boomerang intention through legacy identification, and the relationship between psychological contract violation and boomerang intention through legacy identification. Thus, attention should be paid to whether there is a cooperative relationship between the current enterprise and the former enterprise in management practice. When there is a cooperative relationship, legacy identification is more inclined to become salient, the relationship between the ex-employee and former intention is more closer (Somaya et al., 2008). In this case, it is more easily to cause their boomerang intention, thus bringing the human resource back to the enterprise. But in a non-cooperative organizational relationship, especially in a competitive relationship (Somaya et al., 2008), employees’ legacy identification will often be suppressed by their current organization (Bardon et al., 2015). At this time, the relationship between ex-employees and the former organization is more alienated, the boomerang employment requires more effort from the former organization. The former organization should distinguish these situations, predict the identification salient of the ex-employees, and adopt corresponding strategies.

### Limitations and Future Research Directions

This study makes some theoretical and practical contributions, yet still has some limitations to be improved. First, based on the social identity theory and from the perspective of legacy identification, this study explains the generation mechanism of boomerang intention, which provides a perspective for future research on the generation mechanism of former employees’ boomerang intentions. Legacy identification is a significant perspective for explaining ex-employees’ intentions and behavior. However, there are alternative perspectives, such as social exchange (Herda and Lavelle, 2011), human mourning (Walsh and Bartunek, 2012), social capital (Somaya et al., 2008; Dokko and Rosenkopf, 2010), social network (Farrow and Yuan, 2011; Carnahan and Somaya, 2013), among others. In the future study, boomerang employment can be explained from other perspectives to gain a comprehensive understanding of the generation process of the ex-employee’ s behaviors and intentions.

Second, in addition to boomerang employment, some studies have confirmed that ex-employees can bring their former organization’s many social resources. For example, business cooperation (Carnahan and Somaya, 2013), corporate image management (Potter, 2004), donation (Clotfelter, 2003; Farrow and Yuan, 2011), convey ideas and information (Godart et al., 2014), knowledge transfer (Koc-Menard, 2009; Corredoira and Rosenkopf, 2010), among others. This is because that ex-employee is in the structural hole between their former organization and current organization, and the strong tie between the employee and organization turned into the weak tie after the turnover event. The ex-employee is a kind of significant social resource of the former organization, which should be another significant aspect of ex-employee studies. However, there are only a few studies in this field and almost no studies from a more micro perspective of organizational behavior. In future studies, researchers can pay attention to ex-employee’s prosocial behaviors.

Thirdly, because employees who leave voluntarily have a higher value than employees who leave passively, we define the research object as employees who leave voluntarily. However, a large number of laid-off employees caused by force majeure still have a high value. For example, the COVID-19 pandemic. When the production was stopped at the early stage of the epidemic, a large number of employees were laid-off or stopped working. After the normalization of the epidemic, all kinds of enterprises began to resume production in an orderly manner. In this case, the value of employees who leave passively is not low. Therefore, in future boomerang employment studies, we should not only pay attention to the who leave voluntarily but also pay attention to those who leave passively.

Fourth, in this study, variables were measured at two times points, however, since all the data was self-rated. There is still a chance to cause common method biases which will be harmful to our data analysis result (Podsakoff et al., 2003). Besides, we also should pay attention to the social desirability when these former employees rated these variables by themselves (Crowne and Marlowe, 1964). Thus, data should be collected from multiple sources, such as their leaders or colleagues.

## Data Availability Statement

The raw data supporting the conclusions of this article will be made available by the authors, without undue reservation.

## Ethics Statement

Ethical review and approval was not required for the study on human participants in accordance with the local legislation and institutional requirements. The patients/participants provided their written informed consent to participate in this study.

## Author Contributions

ZT, QY, SQ, and YL: research design and revising the article. ZT: data collection and writing of the original draft. ZT and SQ: data analysis. All authors contributed to the article and approved the submitted version.

## Conflict of Interest

The authors declare that the research was conducted in the absence of any commercial or financial relationships that could be construed as a potential conflict of interest.

## Publisher’s Note

All claims expressed in this article are solely those of the authors and do not necessarily represent those of their affiliated organizations, or those of the publisher, the editors and the reviewers. Any product that may be evaluated in this article, or claim that may be made by its manufacturer, is not guaranteed or endorsed by the publisher.
